# Supplementary material for: Hospital pharmacists’, doctors’ and nurses’ perceptions of intra- and inter- professional communication in the context of electronic prescribing and medication administration systems: A qualitative study
Source: PLoS One. 2023 Nov 30;18(11):e0294714. doi: 10.1371/journal.pone.0294714 (PMC10688685; doi:10.1371/journal.pone.0294714)
Supplement: S2 File — (DOCX) [file pone.0294714.s002.docx]

**Additional file 2 – Topic guide for focus groups with pharmacists**

**An exploration of hospital pharmacists’, doctors’ and nurses’ perceptions of intra- and inter-professional communication and electronic prescribing and medication administration systems in an in-patient setting: a qualitative study**

**Pharmacist focus group questions**

Welcome and thank you for taking your time out today to participate in our study. The aim of this focus group is to gain an insight into pharmacists’ experiences and opinions of paper **and/or** electronic prescribing and medication administration (EPMA) systems in use and the impact they have had on your communication with other pharmacists, pharmacy technicians, doctors and nurses. As you have already been informed, this discussion will be audio recorded.

The first series of questions are to help introduce you to one another. Can you please share:

1. Number of years qualified
2. Speciality/band
3. Previous/current experience with a paper based/EPMA administration system

The second series of questions are around what methods you currently have available to communicate information between:

1. Pharmacist – pharmacist,
2. Pharmacist – pharmacy technician
3. Pharmacist – doctor,
4. Pharmacist – nurse (and vice versa)

What kind of information would you typically exchange with another…

1. pharmacist,
2. pharmacy technician
3. doctor,
4. nurse

…on a day to day basis and what method of communication do you use for the different information exchanges? *[Prompts – paper vs. electronic, does it depend on what you’re communicating or the urgency?]*

How might you use an electronic prescribing and medication administration system to communicate information to other pharmacists and other healthcare professionals (HCPs) such as pharmacy technicians, doctors and nurses? (**For non-EPMA site**)/How do you use the current EPMA system to communicate information with pharmacists and other HCPs such as pharmacy technician, doctors and nurse, and how do they use the system to communicate with you? Are there other uses you could imagine putting it to, or ways that you wish your colleagues would use it (**For EPMA site**)

In your experience (**For EPMA site**) what are the (perceived - **For non-EPMA site**) advantages and disadvantages of using an electronic prescribing and medication administration system to communicate information to other pharmacists and HCPs such as pharmacy technicians, doctors and nurses? **[Get participants to write these on post it notes (different colours for advantages and disadvantages) and share after 5 mins]**

Finally, do you have anything else you would like to share regarding communication between pharmacists, pharmacy technicians, doctors and nurses?

Thank you for taking the time to participate in this focus group.
