# Supplementary material for: Hospital pharmacists’, doctors’ and nurses’ perceptions of intra- and inter- professional communication in the context of electronic prescribing and medication administration systems: A qualitative study
Source: PLoS One. 2023 Nov 30;18(11):e0294714. doi: 10.1371/journal.pone.0294714 (PMC10688685; doi:10.1371/journal.pone.0294714)
Supplement: S3 File — (DOCX) [file pone.0294714.s003.docx]

**Additional file 3 – Topic guide for semi-structured interviews with medical and nursing staff**

**An exploration of hospital pharmacists’, doctors’ and nurses’ perceptions of intra- and inter-professional communication and electronic prescribing and medication administration systems in an in-patient setting: a qualitative study**

**Semi-structured interview questions for medical and nursing staff**

Welcome and thank you for taking your time out today to participate in our study. The aim of this interview is to gain an insight into your experiences and opinions on paper **and/or** electronic prescribing and medication administration (EPMA) systems in use and the impact they have had on your communication with pharmacists.

The first series of questions are to gather some information about yourself and your experience with paper/electronic prescribing and medication administration systems.

1. Number of years qualified……………………………………………………………
2. Speciality…………………………………………………………….………………...
3. Previous/current experience with a paper based/electronic prescribing and medication administration system……………………..………………………….
4. Do you liaise with a pharmacist for medication related queries? ………………
5. Do you liaise with a pharmacy technician for medication related queries?……….

The second series of questions are around methods of communication between you and your pharmacist colleagues.

1. What kind of information would you typically exchange with another doctor and nurse on a day to day basis?
2. What methods are available for you to communicate information to another doctor and nurse?
3. What kind of information would you typically exchange with a pharmacist/pharmacy technician on a day to day basis?
4. What methods are available for you to communicate information to a pharmacist/pharmacy technician?
5. You just listed different types of information and methods you use to communication with a pharmacist/pharmacy technician, why do you choose different method of communication for different information exchanges?
6. How effective do you think these methods are in communicating these information exchanges and why?
7. How could an electronic prescribing and medication administration system be used to communicate information to pharmacists/pharmacy technicians? (**For non-EPMA site**) /How could the current EPMA system be utilised to better communicate information with pharmacists/pharmacy technicians? (**For EPMA site**)
8. What are the (perceived – **for non-EPMA site**) advantages of using an electronic prescribing and medication administration system to communicate information to pharmacists/pharmacy technicians?
9. What are the (perceived – **for non-EPMA site**) disadvantages of using an electronic prescribing and medication administration system to communicate information to pharmacists/pharmacy technicians?

Finally, do you have anything else you would like to share regarding your communication with pharmacists (or using an EPMA system as a method to communicate with pharmacists/pharmacy technician – **for EPMA site**)?

Thank you for taking the time to participate in this interview.
