# Supplementary material for: Hospital pharmacists’, doctors’ and nurses’ perceptions of intra- and inter- professional communication in the context of electronic prescribing and medication administration systems: A qualitative study
Source: PLoS One. 2023 Nov 30;18(11):e0294714. doi: 10.1371/journal.pone.0294714 (PMC10688685; doi:10.1371/journal.pone.0294714)
Supplement: Attachment — Submitted filename: Response to reviewers.docx [file pone.0294714.s004.docx]

PONE-D-23-05914

Hospital pharmacists’, doctors’ and nurses’ perceptions of intra- and inter- professional communication in the context of electronic prescribing and medication administration systems: a qualitative study

PLOS ONE

| **Comment** | **Response** |
| --- | --- |
| ***Editor comments*** | |
| In addition to addressing the comments of reviewer 1 please could you also add in some more detail surrounding your reflexive thematic analysis. For example how did you move from codes to themes, what decisions had to be made during this process with regards to developing or removing the codes that you grouped together? | We have added more details to the relevant section of the paper. |
| Also on page 8 line 169 you say that differences in interpretation were resolved through discussion. However, this approach goes against one of the principles of reflexive TA that you are not looking to find a consensus and the use of multiple researchers is more for adding deeper interpretation and richer meaning. In light of this, please rephrase or amend this sentence. | Thank you for highlighting that the sentence concerned gave the incorrect emphasis. We have reworded it accordingly: “Any differences were addressed through discussion to reach a deeper understanding of the data and its interpretation”. |
| Please ensure that your manuscript meets PLOS ONE's style requirements, including those for file naming. The PLOS ONE style templates can be found at  <https://journals.plos.org/plosone/s/file?id=wjVg/PLOSOne_formatting_sample_main_body.pdf>  [https://journals.plos.org/plosone/s/file?id=ba62/PLOSOne_formatting_sample_title_authors_affiliations.pdf](https://gbr01.safelinks.protection.outlook.com/?url=https%3A%2F%2Fjournals.plos.org%2Fplosone%2Fs%2Ffile%3Fid%3Dba62%2FPLOSOne_formatting_sample_title_authors_affiliations.pdf&data=05%7C01%7Cbryony.franklin%40nhs.net%7Ccb31168d62ee4a00a36708db5b1f5600%7C37c354b285b047f5b22207b48d774ee3%7C0%7C1%7C638203962368109545%7CUnknown%7CTWFpbGZsb3d8eyJWIjoiMC4wLjAwMDAiLCJQIjoiV2luMzIiLCJBTiI6Ik1haWwiLCJXVCI6Mn0%3D%7C3000%7C%7C%7C&sdata=FpVM7lA0UY%2BSvgY27%2F3RGtY0pMKDV8BEQAiWCk5D1t0%3D&reserved=0) | We have checked the formatting requirements including the naming conventions, and have named the files accordingly. |
| 2. Thank you for stating the following in the Competing Interests section:  “We have read the journal's policy and the authors of this manuscript have the following competing interests: SMS was part funded by Cerner Limited. Cerner Limited is a vendor of an electronic prescribing system.”  Please confirm that this does not alter your adherence to all PLOS ONE policies on sharing data and materials, by including the following statement: "This does not alter our adherence to PLOS ONE policies on sharing data and materials.” (as detailed online in our guide for authors http://journals.plos.org/plosone/s/competing-interests).  If there are restrictions on sharing of data and/or materials, please state these. Please note that we cannot proceed with consideration of your article until this information has been declared.  Please include your updated Competing Interests statement in your cover letter; we will change the online submission form on your behalf. | We have added the statement as requested - although we were unable to find any reference to this on the link provided.  We have also updated the covering letter accordingly |
| 3. Please note that in order to use the direct billing option the corresponding author must be affiliated with the chosen institute. Please either amend your manuscript to change the affiliation or corresponding author, or email us at plosone@plos.org with a request to remove this option. | The issue is that I have two accounts, one in my UCL affiliation [bryony.deanfranklin@ucl.ac.uk](mailto:bryony.deanfranklin@ucl.ac.uk) and one in my NHS affiliation [bryony.franklin@nhs.net](mailto:bryony.franklin@nhs.net). This paper was submitted via my NHS affiliation – is it possible to merge the two accounts? |
| 4. In your Data Availability statement, you have not specified where the minimal data set underlying the results described in your manuscript can be found. PLOS defines a study's minimal data set as the underlying data used to reach the conclusions drawn in the manuscript and any additional data required to replicate the reported study findings in their entirety. All PLOS journals require that the minimal data set be made fully available. For more information about our data policy, please see http://journals.plos.org/plosone/s/data-availability.  Upon re-submitting your revised manuscript, please upload your study’s minimal underlying data set as either Supporting Information files or to a stable, public repository and include the relevant URLs, DOIs, or accession numbers within your revised cover letter. For a list of acceptable repositories, please see http://journals.plos.org/plosone/s/data-availability#loc-recommended-repositories. Any potentially identifying patient information must be fully anonymized.  Important: If there are ethical or legal restrictions to sharing your data publicly, please explain these restrictions in detail. Please see our guidelines for more information on what we consider unacceptable restrictions to publicly sharing data: http://journals.plos.org/plosone/s/data-availability#loc-unacceptable-data-access-restrictions. Note that it is not acceptable for the authors to be the sole named individuals responsible for ensuring data access.  We will update your Data Availability statement to reflect the information you provide in your cover letter. | In relation to the minimal data set, our ethics approval precludes sharing of the transcripts. Specifically, in the ethics application, we indicated "no" to the question "Will the data be archived for use by other researchers"  This would therefore preclude archiving the full transcripts of the interviews and focus groups, although we would be happy to provide further examples of relevant quotes etc if required.  I would therefore be grateful for your advice as to how we should proceed in this case. I emailed the PLOS ONE office on 6 June and then again on 28 June to ask for advice on this issue, but have had no reply. Since the resubmission deadline is today, I am therefore submitting the paper now, and would be grateful if you can advise further. |
| 5. Please review your reference list to ensure that it is complete and correct. If you have cited papers that have been retracted, please include the rationale for doing so in the manuscript text, or remove these references and replace them with relevant current references. Any changes to the reference list should be mentioned in the rebuttal letter that accompanies your revised manuscript. If you need to cite a retracted article, indicate the article’s retracted status in the References list and also include a citation and full reference for the retraction notice. | We have checked this information and have not cited any papers that have been retracted as far as we are aware. |
| While revising your submission, please upload your figure files to the Preflight Analysis and Conversion Engine (PACE) digital diagnostic tool, [https://pacev2.apexcovantage.com/](https://gbr01.safelinks.protection.outlook.com/?url=https%3A%2F%2Fpacev2.apexcovantage.com%2F&data=05%7C01%7Cbryony.franklin%40nhs.net%7Ccb31168d62ee4a00a36708db5b1f5600%7C37c354b285b047f5b22207b48d774ee3%7C0%7C1%7C638203962368109545%7CUnknown%7CTWFpbGZsb3d8eyJWIjoiMC4wLjAwMDAiLCJQIjoiV2luMzIiLCJBTiI6Ik1haWwiLCJXVCI6Mn0%3D%7C3000%7C%7C%7C&sdata=vnO7QFrmUk0kZCtogvHaDMpXMT3naEToEvLe4%2Ff1Lo0%3D&reserved=0). PACE helps ensure that figures meet PLOS requirements. To use PACE, you must first register as a user. Registration is free. Then, login and navigate to the UPLOAD tab, where you will find detailed instructions on how to use the tool. If you encounter any issues or have any questions when using PACE, please email PLOS at [figures@plos.org](mailto:figures@plos.org). Please note that Supporting Information files do not need this step. | Our paper does not include any figures. |
| ***Reviewer 1 Comments*** | |
| This is an interesting, important and well presented piece of research throwing new light on patient safety and professional communication issues. | Thank you for your positive feedback. |
| The data was collected five years ago and may not reflect current professional or technical practice. It would be helpful to have some understanding of any major systems changes that may have come about in the interim and also to reflect on how the current situation may be further researched. The major issue of Covid in the interim may be of significance - did this have an impact on prescribing systems? It certainly will have impacted on models of professional communication... | Thank you for raising this important point – we agree and have added this as a further limitation as follows: “Finally, the data were collected prior to the COVID-19 pandemic, which may have resulted in changes in communication practices and the use of ePMA. For example, staff may now be more likely to work remotely, introducing additional communication challenges.”  In the section on future work we have added “…and any impact of remote working becoming more prevalent following the COVID-19 pandemic” |
| For those who are not familiar with NHS systems, it would be useful to briefly indicate the main features / differences of the ePMA systems - e.g. recording only, advisory, highlight risk issues, detect errors etc. | Thank you for suggesting this. We have added the following brief context: “The Cerner ePMA system was part of a wider electronic health records system; the JAC system did not include electronic health records but was instead integrated with the pharmacy dispensing system. Both allowed prescribing and recording of medication administration and included basic decision support.” |
| An impressive number of participants were involved but there is significant variation between professions, grades and sites in terms of who took part; the absence of consultants from two hospitals is striking. This may reflect the more involved role of junior doctors but it raises issues about the involvement, understanding and experience of those who are often making the prescribing decisions rather than simply recording them. Is there a place for further research here? | We already included some reference to this in the limitations section; i.e., “Most of the doctors who took part were less than four years qualified and so the views of more experienced doctors may not have been captured, although junior doctors often have the most interaction with ePMA systems as they do most of the prescribing for hospital inpatients in UK hospitals” – we have now also added in the text “such as consultants” to emphasise this point. We have also added the recommendation that future work also includes “the perspectives of more senior doctors”. |
| The methodology and study guide are appropriate - however, the balance of the data seems to reflect the disadvantages or possible improvements needed for the systems. This is understandable in this research framework but there is limited information on the reported strengths / advantages of the systems - the authors might consider if such information might be explored. | The reviewer is correct that the study participants tended to focus on the disadvantages of ePMA systems in relation to communication, in spite of the topic guides asking about both advantages and disadvantages. However in the light of the reviewers’ comments, we revisited our data and identified aa further advantage of ePMA, around supporting more complete documentation, which we have now added into the paper since this links to communication. We have also added a comment in the discussion section highlighting how participants tended to focus on the disadvantages. |
| The discussion highlights important experience among HCPs which might not otherwise have emerged in other research settings. Broader exploration of further and up-to-date research opportunities might be of value. | In the section on future work we have added “…and any impact of remote working becoming more prevalent following the COVID-19 pandemic” to highlight a reason why more up to date work may be helpful. |
| ***Reviewer 2 Comments*** | |
| Dear authors, thank you for opportunity to review this manuscript. In general, I consider the paper to be really well written. Background and methodology are clearly explained, results well presented. I believe the manuscript meets expected standards and I recommend the article for publishing. | Thank you for your positive feedback. |
